# Ergot in Obstetric Practice

**Published:** 1889-07

**Authors:** 


### ERGOT IN OBSTETRIC PRACTICE.

Professor John Spottswood Wellford of Richmond, Va., furnishes us the following in answer to our request for his views concerning the proper use of ergot in obstetric practice. He says:

"Without going into the theoretical views of the action of ergot, there are some points which are of primary importance in a practical view. The normal uterine contractions are intermittent and of comparatively short duration. The ergotic contractions are constant and steady, and almost tetanic. Consequently the child is subjected to persistent pressure, which, if continued for some time, produces death. This fact suggests one invariable rule for its use with regard to the child. Never give ergot until every impediment is removed for the speedy delivery of the child. Of course where the life of the mother is so imperilled that the practitioner is justified in running some risk with the child, then it would be necessary, and ergot would be advisable. But, under most circumstances, the use of the forceps would be then preferable.

"In consequence of these views, which could be very much enlarged, the judicious use of ergot should be confined to the last stage of labor, but regulated by the condition of the patient. In a multipara it might be useful as soon as the os is dilated, while in a primipara it should not be given until the second stage, and not then unless the soft parts are thoroughly relaxed. When I am satisfied that the parts are prepared for the speedy delivery, and there is no probability of any delay, I would have no hesitation in giving ergot until I produced its specific effect.

"But there is one object to be accomplished by ergot which is not fully appreciated by some authorities—that is, the prevention of the post-partum hemorrhage and after-pains. Chloroform, which may, and I think, should be, given in nearly every case of labor, is liable to the objection that it may increase the tendency to hemorrhage. In every case, immediately after the expulsion of the child, the womb, as it were, exhausted by the throes of labor rests for a longer or shorter time to repair its strength; and again, this inertia may occur after the expulsion of the placenta when the bleeding orifices of the torn vessels at the insertion of the placenta are still patulus for the want of uterine contraction. When profoundly anæsthetized, this condition may be injuriously prolonged by the effect of the chloroform. To guard against such a result, I always give the ergot when I think that by the time it could be absorbed, and its specific effect be produced, the child would be either born, or so nearly so, that no injurious pressure could be exercised. By this means the expulsion of the placenta is hastened, the danger of adhesion is prevented, and the womb is cleared of all clots or pieces of the secondines, and firm contractions are permanently established. The danger of post-partum hemorrhage is removed, and the womb being thus contracted, the multipara is placed in the same condition as the primapara, and she rarely suffers, after the effect of the ergot has passed off, with any after-pains, and the chances of complete involuticn are very much increased. Chloroform increases the relaxation, relieves the pain and sufferings both mental and physical, and, by the improved condition of the patient, it diminishes the probabilities of infection. Ergot given in the way I suggest hurries the progress of the case, prevents any possible injurious effect of the choroform, produces immediate closure and sealing of the patulous blood vessels, bars the entrance of septic material, and secures, without the occurrence of after-pains, the subsequent involution. For these reasons both articles should really be used in nearly every case, as a routine practice, for the reason that normal labor is a physiological, not a pathological process, and thereby permits and requires a routine.

"The dose necessary to secure the objects named is usually one teaspoonful of a reliable Fluid Extract mixed with about an ounce of water, and repeated if necessary. Of course when the labor is lingering, after full relaxation of the soft parts has taken place; when the pains are suspended, or the patient is exhausted by the long continuance of the labor, or after the occurrence of post-partum hemorrhage, the full effect of the ergot should be produced and the doses repeated at short intervals."—*Practice.*

